# Alterations in Content and Localization of Defensins in Rat Ileum and Jejunum Following Ischemia-Reperfusion. Specific Peptides, in Specific Places, for Specific Jobs?

**Published:** 2011-02-23

**Authors:** Rosemary A. Kozar, Rachel J. Santora, Brian J. Poindexter, Stephen M. Milner, Roger J. Bick

**Affiliations:** ^a^Department of Surgery; ^b^Pathology, University of Texas Medical School at Houston, Tx; ^c^Johns Hopkins Burn Center, Johns Hopkins University School of Medicine, Baltimore, Md

## Abstract

**Objective:** To determine alterations in quantities and distributions of natural antimicrobials following ischemia-reperfusion injury. We hypothesized that these compounds would be upregulated in areas of small intestine where changes in permeability and cellular disruption were likely and where protective mechanisms would be initiated. **Methods:** Rats with ischemia-reperfusion underwent superior mesenteric artery clamping and reperfusion. Shams were subjected to laparotomy but no clamping. Ileum and jejunum were harvested and sectioned, and subjected to fluorescence deconvolution microscopy for determinations of content and localization of rat beta defensins, 1, 2, 3; rat neutrophil protein-1; and cathelicidin LL-37. Modeling was performed to determine cellular location of antimicrobials. **Results:** Ischemia-reperfusion increased neutrophil defensin alpha (RNP-1) in jejunum; rat beta defensin 1 was increased 2-fold in ileal mucosa and slightly reduced in jejunal mucosa; rat beta defensin 2 was reduced by ischemia-reperfusion in ileum, but slightly increased in jejunum; rat beta defensin 3 was concentrated in the muscularis externa and myenteric plexus of the jejunum; ischemia-reperfusion did not alter cathelicidin LL-37 content in the small intestine, although a greater concentration was seen in jejunum compared with ileum. **Conclusion:** Ischemia-reperfusion injury caused changes in antimicrobial content in defined areas, and these different regulations might reflect the specific roles of jejunum versus ileum.

Intestinal mucosa is very susceptible to ischemia-reperfusion (I/R) initiated changes that are often seen after shock and small-bowel transplantation. Previous work has shown major consequences of intestinal I/R injury, such as changes in gene expression patterns, altered levels of component calcium handling proteins, and cytoskeletal disruption.^1^^-^3 Our hypothesis was that as areas and cells of the small intestine were traumatized, protective mechanisms would be initiated, and that one of these mechanisms would be increases in defensin content in specific layers of the tissue.^4^

Sloughing of cells following substantial blebbing due to prolonged ischemia has been reported as a nonreversible injury, such that rates and amounts of cell destruction need to be determined to understand the temporal aspects of cell loss and necrosis in order to initiate protective, rather than recovery, therapies.^5^ Hemorrhage in the mesentery causes increased concentrations of not only proinflammatory cytokines, but also an α-defensin product, while intestinal I/R results in increased levels of β-defensin 2, possibly as a protective factor to combat acute lung injury due to increased levels of tumor necrosis factor α.^6^,^7^ It is evident that defensins have a significant role in the protection of various tissues including the intestine.^8^ However, it has also been reported that enteric defensins are intimately involved in microbial regulation as well as having individual, distinct roles in defined areas of the gastrointestinal tract, a finding we have seen before and also report here.^9^ Furthermore, we report differences regarding changes in peptide content due to I/R, not unlike those observations regarding β-defensin induction due to numerous diseases and hemorrhagic shock.^4^,^10^^-^14

Our previous studies investigating heart failure led us to believe that defensins were largely associated with vascular endothelial cells, a localization noted by others.^13^ It is not surprising, therefore, that the images acquired in this study also indicate an intimate relationship of peptides with vascular elements of the small intestine. We studied these particular defensins as we have seen concentration and localization changes in these peptides associated with burn injury, and others have reported alterations in β-defensin expression with intestinal problems,^7^,^8^,^11^,^12^ and because of a recent publication regarding mucosal immunity.^8^

The results suggest that not only are defensins in a position to combat the invasion of bacteria from the intestinal lumen but also to counteract circulating microbes and, as such, have at least a role in a 2-pronged defense.

## METHODS

All chemicals were purchased from Sigma Chemical Corp, (St Louis, Mo) except where stated, and are of the highest grade available.

### Animal Surgery

All procedures and protocols were approved by the University of Texas Houston Medical School Animal Welfare Committee. Male Sprague-Dawley rats weighing 250 to 300 g were obtained and housed individually. They were kept at room temperature (25°C) with alternating 12-hour light/dark cycles. Animals were fed standard rat chow and water ad libitum during a minimum acclimation period of 5 days. Each animal was fasted with free access to water overnight before laparotomy. Operative procedures were performed using sterile techniques under general anesthesia with isoflurane-inhaled anesthetic.

A midline laparotomy was made, and the gut I/R was carried out by superior mesenteric artery occlusion for 60 minutes. After removal of the clamp, the incision was closed and the rats were allowed to awaken and were observed for 6 hours. Sham animals underwent an identical procedure but without placement of the clamp on the superior mesenteric artery. At the end of 6 hours, animals were sacrificed under isoflurane anesthesia by exsanguination, and full thickness segments of jejunum 5 cm distal to the ligament of Treitz and ileum, 2 cm proximal to the cecum, were harvested. These measurements were always used to assure that tissues sectioning was consistent.

### Tissue Preparation

After surgery, tissue samples from ileum and jejunum were removed immediately, embedded in sucrose-based O.C.T. compound (Tissue-Tek, Torrance, Calif) and frozen on dry ice. Processing of samples and methodology for fluorescence deconvolution microscopy have been presented and discussed in detail.^2^,^3^,^11^ Briefly, frozen sections were cut to a thickness of 12 ± 3 µm with a cryotome (Microm HM 505 E, Microm Laboratories, Walldorf, Germany) and placed on 18-mm, acid-cleaned, glass cover slips (Fisher, Pittsburgh, Pa), coated with poly-L-lysine. Sections were rinsed with cold phosphate buffered saline, fixed in 3.7% paraformaldehyde (Tousimis Research, Rockville, Md) at room temperature, and rinsed 5 times with phosphate buffered saline at room temperature. Cover slips were inverted and floated on 10% goat serum for 1 hour at 37°C to reduce nonspecific antibody binding, then antibodies against the defensins β-defensin 1, β-defensin 2, neutrophil defensin NP-1 (Alpha Diagnostic, San Antonio, Tex), β-defensin 3 (Novus Biologicals, Littleton, Colo), cathelicidin LL-37 (Hycult Biotechnology b.v., Uden, the Netherlands), and smooth muscle actin (Sigma, St Louis, Mo) were diluted 1:100 in 10% goat serum and incubated with tissue sections for 45 minutes at 37°C. After rinsing the coverslips in 0.05% Tween20, fluorescently tagged secondary antibodies (Molecular Probes, Eugene, Ore) were added and the sections were incubated for 30 minutes at 37°C. Finally, F-Actin and the nuclei were simultaneously stained with phallacidin and 4′, 6-diamidino-2-phenylindole (DAPI), respectively (Molecular Probes, Eugene, Ore) for 15 minutes at room temperature. Cover slips were mounted onto glass slides with Elvanol antifade (DuPont, Wilmington, Del) and attached with nail polish.

### Reconstructive Microscopy (Deconvolution)

Three portions of ileum and jejunum were taken from each of 3 I/R and 3 sham animals, and 3 sections from each portion were taken. Three scans were performed on each section giving a total of 81 data acquisitions per group. Specimens were scanned with an Applied Precision DeltaVision (Issaquah, Wash) system fitted with an Olympus IX 70 inverted microscope employing a 100-W mercury arc lamp for illumination (Olympus America, Melville, NY), and excitation/emission filter sets (Chroma Technology Corp, Brattleboro, Vt) specific for each of the fluorescent antibodies. Filter-set combinations were set for each probe as follows: DAPI (nucleus), 340-nm excitation filter (band-pass of 20 nm), and an emission filter of 390 nm (band-pass of 20 nm); Phallicidin (F-Actin), excitation filter of 488 nm (band-pass 10 nm), and an emission filter of 520 nm (band-pass 25 nm); defensin antibodies, excitation filter of 555 nm (band-pass 28 nm), and an emission filter of 617 nm (band-pass 73 nm); and smooth muscle actin, excitation filter of 640 nm (band-pass 20 nm), and emission filter of 685 nm (band pass of 40 nm). Image scans for each probe were acquired in series at a step size of 0.35 µm with a Sony Interline CCD camera (Sony Corp., New York, NY). At least 30 sections were scanned per sample for each probe. Deconvolution and image analysis were performed by transferring the data sets to a Linux/RedHat workstation employing SoftWoRx software (Applied Precision, Issaquah, WA) using an algorithm experimentally produced on the system from the convolution of a point-spread function to differentiate and reduce extraneous light or scattered light captured by the camera, derived by scanning a 0.1 µm fluorescent bead (Molecular Probes, Eugene, Ore) 4 µm above and 4 µm below the plane of focus. The resulting point-spread function is Fourier transformed into an optical transfer function that uses the data to produce images with a higher signal-to-noise resolution of the probe emission patterns. All data sets are subjected to 5 to 15 deconvolution iterations and then analyzed, reconstructed, volume rendered, and modeled. Subtraction of background fluorescence and change of intensity gain were optimally set for each emission.

An image projection is produced by stacking each of the individual z-sections of the acquisition into one image, resulting in a 3-dimensional image with overlaid colors. Volume rendering uses these z-section stacks and rotates them about the X or Y plane and a 3-dimensional, computer-generated, virtual model of the fluorescence emission patterns is produced to view relative positions of each emission/probe and determine the localization of defensins to specific cells types and tissue areas.^2^ The 3-dimensional model is fully interactive and can be rotated in any plane for viewing at any angle or perspective.

## RESULTS

**Figure 1:**
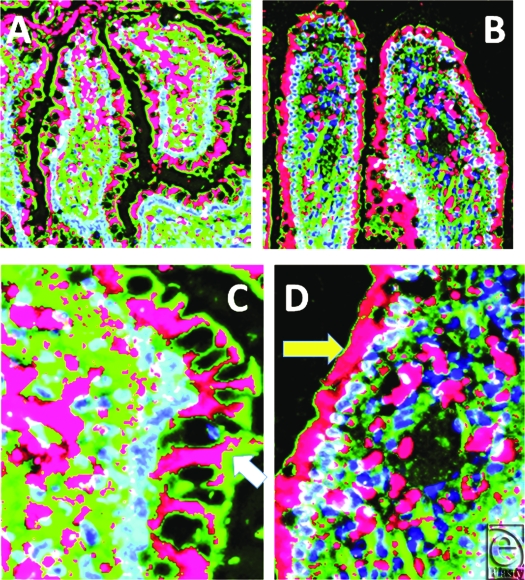
Comparison of the content and localization of rat beta defensin 1 in the ileum of control (*panel A*) and ischemia-reperfusion (*panel B*) animals. Panels C and D are cut-out sections at a higher magnification to show the extent of antimicrobial “filling” of the surface enterocytes. Also of note is the defensin (red) associated with the central area of the villus. Magnification (*panels A and B*) X 400; magnification (*panels C and D*) X 900. F-actin is green, nuclei are blue. Deconvolution iterations 15.

Figure 1 includes 2 pairs of images (control-shams on the left, I/R tissues on the right) and compares and contrasts the content and localization of rat beta defensin (RBD)-1 in the ileum of control (A) and I/R (B) animals (magnification X 400). Panels C and D are cut-out sections at a higher magnification (X 1200) to show that enterocytes contained more peptide after I/R, with high concentrations of RBD-1 in and around the central lacteal area of these villi both before and after I/R injury.

**Figure 2:**
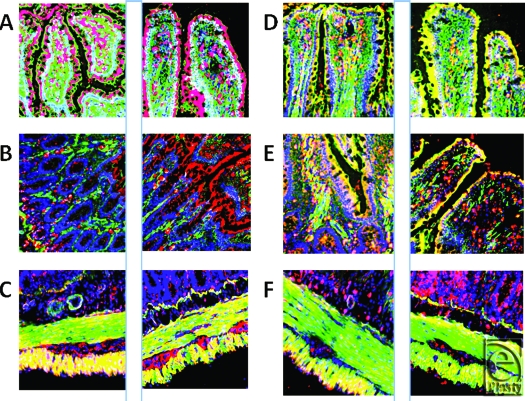
This figure demonstrates the specificities with regard to both concentration changes and localizations of various antimicrobials in the ileum. Each pair of images (*panels A through F*) shows the sham tissue on the left and the ischemia-reperfusion image on the right. The first 2 panels (*A*) are images used to produce Figure 1. More rat beta defensin (RBD)-1 is seen in the apical and middle regions of the villi. Panel B again shows RBD-1 (red), but this time in the base/neck region of the villi. Panel C shows RBD-3 before and after ischemia-reperfusion demonstrating a decrease in peptide in the outer, longitudinal muscularis externa and a small increase in the inner, circular layer. Panel D shows RBD-2 increase in enterocytes due to I/R and a decrease in perilacteal concentration. Panel E shows RNP-1 throughout the epithelial layer of both sham and ischemia-reperfusion samples; panel F clearly demonstrates a reduction of cathelicidin in the outer, longitudinal muscularis externa, but an increase in the lower, glandular aspects of the mucosa (magnification X 400).

To further show the regulations and specificities, Figure 2 includes 6 pairs of images (shams on the left and I/R samples on the right). In each of the panels, the antimicrobial peptide is shown in red (orange/yellow where colocalization occurs). Cell nuclei are blue and F-actin is green. The first pair (panel A) are the images from Figure 1, but of note here is the high concentration of RBD-1 seen in the lower portion of the villi in the I/R sample. Panel B also shows RBD-1, but in the lower portion of the mucosa, where the crypts meet the glands. Note the increased amount of (red) peptide following I/R. Panel C is of RBD-3, located at an extremely high concentration in the longitudinal layer of the muscularis externa, but greatly reduced following I/R. However, this loss is somewhat compensated for by an increase in the inner, circular layer of the externa and maybe even in the myenteric plexus. Panel D demonstrates that I/R injury shows a trend of increasing RBD-2 in luminal epithelial cells in jejunum as seen in this pair of images, but not in ileum. Overall, the values are not significantly different; however, as stated, the trend within pairs is for an increase in staining intensity as shown here (13300 ± 7889 vs 16123 ± 7066; sham vs I/R pixels). Panel E shows that there was no change (18938 ± 8607 vs 18245 ± 7168; sham vs I/R; pixels) when comparing concentrations of RNP-1. The final pair of images, panel F, shows that LL-37 was also found predominantly in the outer muscularis externa, as was RBD-3, and that the amount was dramatically reduced by I/R, while this loss was compensated for by a dramatic increase in the lower gland cells, in the area one would expect to find Paneth cells, of the mucosa.

Overall, even though total peptide content of small intestine samples might not appear to change in many sections, changes in specific components of full thickness sections did indeed show dramatic differences. Also, a general rule could be applied in that the ileum showed an increase, or at least no decrease, in peptide content following I/R, while the jejunum revealed slight decreases.

**Figure 3:**
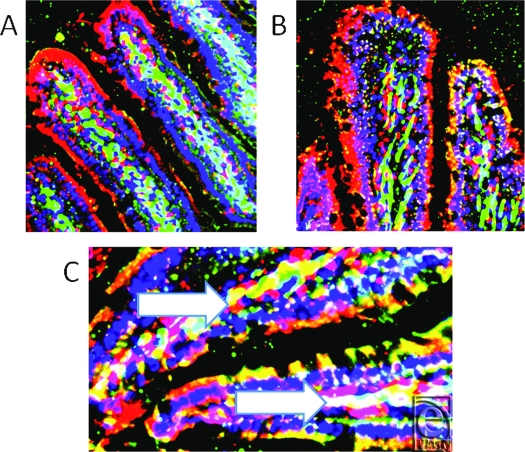
Panel A shows very high concentration of rat beta defensin 2 (red) in specific areas of this sample of I/R jejunum, while in I/R ileum (*image B*), rat beta defensin 2 is seen in surface epithelium, neck region and lower glands. Panel C shows high-power (magnification X 1200) cut out from panel A to demonstrate the central concentrations of peptide that show as both red and white areas, the latter due to colocalization with smooth muscle actin of the small vessels (magnification X 400).

Panel A in Figure 3 demonstrates the very high concentration and specific localization of RBD-2 (red) in this sample of I/R jejunum, while in I/R ileum (image B), the RBD-2 is found throughout the epithelium, even into the neck and lower glands. Panel C is a high-power image cut out of I/R jejunum, to demonstrate the central concentrations of peptide, (identifiable as both red and white due to colocalization with green probe) localized with the smooth muscle actin of the small vessels (magnification X 1200).

**Figure 4:**
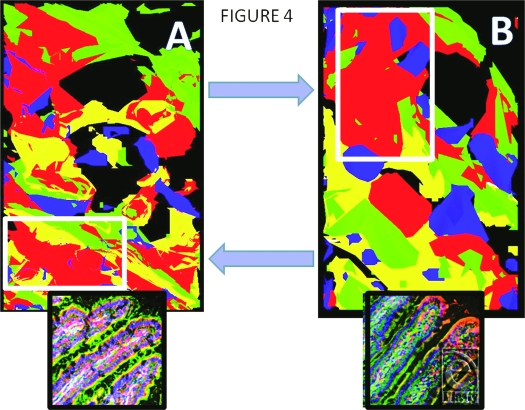
Models constructed by image stacking, as described under “Methods” section, of sham jejunum (*panel A*) and ischemia-reperfusion jejunum (*panel B*) to detail changes in specific areas. The upper blue arrow points to increased rat beta defensin 2 (red) in this enterocyte, adjacent to a goblet cell, following ischemia-reperfusion; the lower blue arrow indicates rat beta defensin 2 in the central-lacteal region of this villus, following ischemia-reperfusion. The small inserts show the stacked images from which the models were generated. Nuclei are blue, F-actin is green (magnification X 400).

Figure 4 shows models constructed by image stacking, as described under “Methods” section, of sham jejunum (panel A) and I/R jejunum (panel B) to show the distinct changes at defined levels. The upper blue arrow points to the increase in RBD-2 (red) in the enterocyte, adjacent to a goblet cell, following I/R, while the lower blue arrow points to the decrease in RBD-2 in the central-lacteal region of these villi, following I/R injury. The small inserts show the stacked images from which the models were generated. Nuclei are blue, F-actin is green.

To show theses specificities, we prepared some cropped images and measured pixel densities to compare against full thickness images, while also comparing tissue, ileum versus jejunum, and peptide content (Table 1). As can be seen, RBD-1 content in ileum was increased following I/R, while it was decreased in jejunum. In contrast, RNP-1 content in jejunum was increased, while in ileum, it remained unchanged. Cropping sections (Table 1, part B) also revealed that localizations such as RBD-3 in ileum was found mostly in the externa (see Fig 2, panel C), while in the sham jejunum, it was very apparent in the myenteric plexus and was decreased following I/R.

## DISCUSSION

Defensins are small cationic peptides that are important components of the innate immune system especially in intestinal defense,^15^^-^18 and there are many reports that defensins have more than just an antitoxin role. Although this report is of a nonfunctional, morphological study, it does support previous findings of specificities among antimicrobial peptides as to their role(s) in protection and we wanted to determine whether antimicrobial peptides were possibly part of the injury process, becoming a barrier against ensuing toxic events. A recent report suggested that human β-defensin 2 has a chemotactic affect with regard to endothelial cells and might well be proangiogenic and this possibility fits nicely with our finding of colocalization of defensins to capillaries and the microvasculature in the lacteals, myenteric plexus, muscularis externa, and adventitia seen in our images.^11^,^19^

Using deconvolution microscopy, we have shown that defensins are in certain cells and areas, and put forward suggestions as to the functional diversity of the antimicrobial peptides. We chose to examine the particular peptides noted in this work because of their continued appearance in the literature concerning intestinal well-being, and although our observations with cathelicidin LL-37 pose a bit of a quandary, this peptide having previously been associated only with epithelial cells of the colon, Termen et al did report the rat cathelicidin rCRAMP being identified in small intestine.^20^^-^22 It is, therefore, evident that multiple mechanisms and compounds are employed to combat bacterial infection and, more importantly, bacterial entry into underlying tissues.

Reported research as to the foci of specific antimicrobials lends evidence to particular functions as suggested by our findings. For example, LL-37 was found in high concentrations in the apical portions of many villi following I/R, possibly pointing to its role in enhanced endothelial proliferation^23^ as well as in cell protection and healing effects.^24^,^25^ The finding of LL-37 in the outer externa, adventitia, and serosa also suggests that it might exert its reported angiogenic effects in these areas, overcoming or delaying necrotic and apoptotic cell death.^24^

It has been noted that an understanding of the mechanisms, roles, and targets of individual antimicrobials in maintaining the delicate balance of retaining endogenous flora versus eliminating exogenous pathogens is necessary to develop therapeutic strategies in treating intestinal ailments and replenishing the endothelium.^25^ A relatively recent review strengthened the need for understanding the mechanisms and cellular locations of the multiple defensins and cathelicidins found in the gastrointestinal tract, noting that advances in new therapies can be made if we do indeed have insights into the roles and mechanisms of these peptides.^26^ A more recent report stressed the “multilayered regulation” of gastrointestinal defense, and our findings of specific geographic locations of inducible defensins and intimate associations with particular cell types, yields a better understanding of potential therapeutic targets in overcoming intestinal inflammation, destruction, and cell loss while adding further understanding to the “biology of defensins and LL-37,” as well as “beginning to clarify the pathophysiology of mucosal and infectious diseases.”^27^^-^31 Further elucidation of regulatory changes associated with chronic inflammatory and debilitating diseases will allow us to not only target appropriate sites and tissues, but will arm us with an understanding of when to initiate specific therapies and even give us less traumatic methods for ensuring intestinal well-being.^30^,^31^ The apparent specific roles for defensins with regard to position and protection at luminal epithelium and endothelium, be that of the vasculature or lining the gut lumen, is a somewhat novel finding, and although publications of vasculature endothelium localization have been forthcoming, that data are because of cancer formation.^32^,^33^

There are concerns with the work. The number of animals was relatively small; however, the number of data acquisition points was large. It is also possible that there is cross-reaction of antibodies and probes, particularly with regard to rat cathelicidin (CRAMP) and its lack of homology with other cathelicidins. However, a previous report did show specific staining.^11^ Future studies should probably use antibodies raised against rat cathelicidin, but this might be difficult to achieve due to financial constraints.

## CONCLUSION

Although this study is observational, the quantity measurements by different methods does show that different peptides reside in different layers of the intestine, and these findings lead us to our conclusions that in diverse tissues, defensins are found in both luminal epithelium and blood vessel epithelium at a time when severe damage to tissue integrity and cell function is imminent. In areas that require increased protection, this is an important factor in times of gastrointestinal tract infection, ischemia, and sepsis and leads us to consider antimicrobial upregulation maneuvers as a means of therapy.

## Figures and Tables

**Table 1:** Comparison of Peptide Content in Specific Layers of the Small Intestine in Ileum Versus Jejunum and Sham Versus I/R Tissues

Tissue	Peptide	Sham Pixels	I/R Pixels	Trend	
Ileum	RBD-1	5377 ± 2181	1028613705	Up*	
Jejunum	RBD-1	1048611751	736611542	Down†	
Ileum	RNP-1	1893818607	1824517168	No change	
Jejunum	RNP-1	1599615295	1959517544	Up‡	
			PART B		
		Sham	Action		Conclusion
Tissue	Peptide	Pixels		Pixels	
Ileum	RBD-3	1662715297	Remove externa	436711778	Much in externa§
Jejunum	RBD-3	1395213446	Remove externa and Auerbach's	616112394	Much in Auerbach's|

I/R indicates ischemia-reperfusion; RBD, rat beta defensin.

*P < .05 I/R versus sham.

†*P* < .05 sham versus I/R.

‡*P* < .10 I/R versus sham.

§*P* < .01 with externa versus without externa.

|*P* < .01 with externa versus without externa.
